# Individual Disaster Preparedness in Drought-and-Flood-Prone Villages in Northwest China: Impact of Place, Out-Migration and Community

**DOI:** 10.3390/ijerph18041649

**Published:** 2021-02-09

**Authors:** Chunlan Guo, Timothy Sim, Guiwu Su

**Affiliations:** 1 World Health Organization Collaborating Centre for Community Health Services, School of Nursing, The Hong Kong Polytechnic University, Hung Hum, Hong Kong, China; 2 Department of Applied Social Sciences, The Hong Kong Polytechnic University, Hung Hum, Hong Kong, China; timothy.sim@polyu.edu.hk; 3 S R Nathan School of Human Development, Singapore University of Social Sciences, Singapore 599494, Singapore; 4 Institute of Geology, China Earthquake Administration, Beijing 100029, China; suguiwu@ies.ac.cn

**Keywords:** disaster preparedness, disaster risk reduction, place, migration, neighbourhood, rural area, Northwest China

## Abstract

Rural communities are generally more vulnerable to natural hazards when compared to urban communities. Moreover, rural communities are diverse and unique in their place, population, agricultural production and culture, which make it challenging for different rural settings to prepare for disasters. There is a little comparison made about the individual disaster preparedness among rural communities with different geographic landforms. In this study, we examined the individual disaster preparedness of rural residents in three drought-and-flood-prone villages with different landforms (plains, loess plateau and mountains) via a cross-sectional self-report structured questionnaire survey conducted in Northwest China. We also adopted an ecological framework to examine the determinants of villagers’ individual disaster preparedness across different dimensions: place, individual sociodemographic factors, family socioeconomic status, hazard adaptations, community and neighbourhood influences. We found that place was a significant factor for disaster preparedness when controlling individual sociodemographic and family socioeconomic factors. The level of preparedness in the plains was higher than both mountains and plateau. Moreover, the villagers who had out-migrated to work reported a higher level of disaster preparedness than did local villagers. In addition, the community and neighbourhood played an important role in determining individual disaster preparedness. This research highlights the needs for tailored community-based disaster risk reduction programs to improve villagers’ knowledge and skills of disaster preparedness.

## 1. Introduction 

Rural areas face different challenges from their urban counterparts pertaining to disaster risks and management [1,2]. Compared to urban areas, rural communities often have a less diversified economy and fewer financial resources to support disaster risk reduction or recovery after disasters [3]. Moreover, low population density and inadequate communication networks may pose more challenges to rural communities in disaster risk reduction (DRR) [1,4]. Additionally, rural areas often lack disaster training programs due to their scarce resources for planning, training, and responding to disasters [4,5]. In fact, rural communities are diverse and unique in their place, population, agricultural production and culture. There is a little comparison of rural communities’ disaster risk and preparedness in different regions with different geographical landforms and environments.

Disaster preparedness is a behaviour, which could reduce the risk of injury and damage, and facilitate a capability for coping with the temporary disruption associated with hazard activity [6]. Disaster preparedness could be categorized into different levels, such as community, institutional, household and individual level. Most of the existing disaster preparedness in rural areas focused on the household level, e.g., [7,8,9,10,11]. However, disaster preparedness at the individual level is also important for rural communities, especially in disaster-prone and under-resourced villages.

Usually, risk and vulnerability are different in different places with different landforms [12,13]. For example, drought is one of the most widespread and destructive hazards over the loess plateau of China [14,15]. However, the plains next to a river or stream encounter a high risk of floods [16]. The livelihood in rural areas usually are more relied on natural resources and most rural residents have higher environmental exposure, which might cause them a higher risk of environmental hazards, such as extreme weather [17] and floods [18]. Therefore, place plays a significant role when discussing disaster preparedness in rural communities.

Existing research has also identified a list of individual sociodemographic factors associated with disaster preparedness, e.g., [19,20,21], which includes migration status [22,23], race and ethnicity [24], income [25], education level [26] and homeownership [27]. Hazard adaption, which is usually constructed by hazard experience, knowledge, risk perception and self-efficacy [28], is another individual inherent factor which may have a significant influence on disaster preparedness [7,29,30,31,32,33,34]. Generally, individuals with previous disaster experiences are more likely than those without to better prepare for disasters [34]. For those who have not experienced such types of disasters, their preparedness will be dependent upon the exposure to other forms of disaster with applicable transferable knowledge, access and participation in drills related to disaster preparedness, as well as communication channels (e.g., social gatherings, newspapers, radio, TV, social media) [35,36]. Furthermore, self-efficacy has a significant and positive impact on preparedness [37]. Higher self-efficacy may also mediate the association between personally experiencing a hazard and taking preventive action [38,39].

Moreover, the socioeconomic status of the family is a significant predictor of whether individual adopts preventive and safety behaviour [40]. For the rural families with a larger agricultural landmass, there is usually more perceived personal fiscal risk with drought and, hence, more preparedness upon drought. However, it is surprising to find that the larger the farmland a rural family possesses, the less likely it will be to take responding measures to drought accordingly in the North China Plain since many of these families cannot afford the costs of agricultural materials and labour forces caused by the drought and they will not choose to take action for a single piece of land [41]. In addition, when more family members work in non-agricultural employment, the less likely the household practice of preparedness to drought because the family economic income is less dependent on agricultural production [41].

Communities can exert considerable influence on the behaviours of individuals, which include behaviours adopted to be prepared for disasters [42]. Usually, the more residents are engaged in their communities and neighbourhood, the more they prepare for hazards [43]. Studying the influences of communities brings to bear holistic, contextual and meaningful information about complex socio-cultural interactions between community members and disaster preparedness [44]. Moreover, studying more than one community provides an opportunity to observe the diverse and unique individual responses to the disaster by places [45]. This was another main rationale for conducting this study.

Therefore, the overall aim of this study is to access the individual disaster preparedness of villagers in northwest rural China and examine the determinants of their individual disaster preparedness across different dimensions: place, individual sociodemographic factors, family socioeconomic status, hazard adaptations, community and neighbourhood influences. The interactions between different determinants will be discussed. In turn, these findings will help to improve DRR program designs to increase individual disaster preparedness in rural communities.

## 2. Method

### 2.1. Site Selection

**Figure 1:**
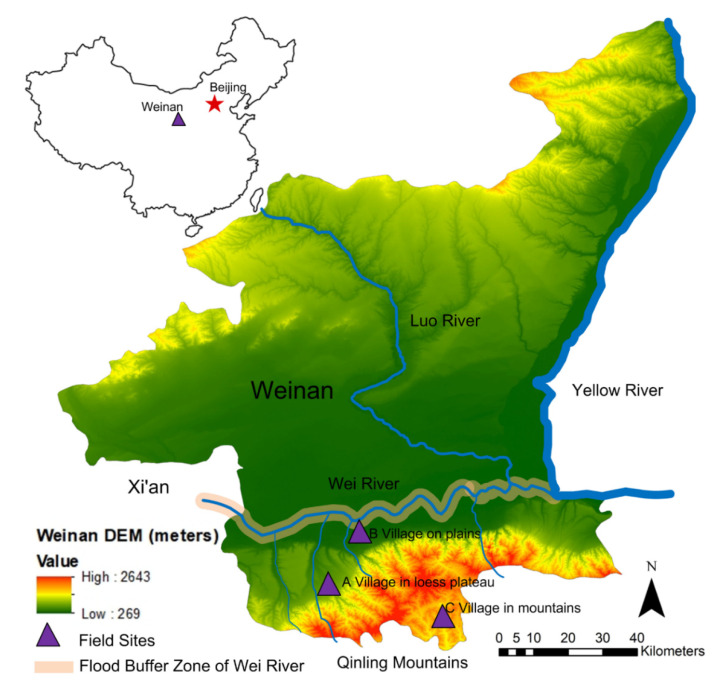
Map of site selection. Source: DEM data was derived from Space Shuttle Radar Topography Mission, NASA [55].

A multi-level population-based stratified approach was adopted to capture a representative sample of rural residents living in poverty with different geographic landforms in Northwest China. Poverty and geographic landforms were two factors controlled for the site selection. Considering the absolute value and proportion of poor individuals in the communities as well as their unique landforms, the study selected one village in loess plateau (A), one on plains (B) and one in the mountains (C) in Weinan City of Shaanxi Province, located in Northwest China, for the questionnaire surveys (Figure 1). It is noted that all three villages were registered in the national poverty reduction project [46,47]. Moreover, due to climate changes, this region faced massive environmental degradation and climatic and hydrological hazards, such as droughts and floods [48,49,50]. The duration of regional meteorological drought events was mainly dominated by 10–20 days, reaching a maximum of 218 days according to the data of Shaanxi Province from 1961 to 2013 [51]. The regional drought events occurred mainly in spring and summer each year [51]. The village on plains (B), which is located next to the Wei River, the largest tributary of the Yellow River, encountered floods frequently [52], such as the disastrous floods that occurred in 2003 [53] and 2011 [54].

The above site selection could largely eliminate the possible influences of distant location (within 50 km from each other), language and dialect (using the *Huazhou* dialect), ethnicity (almost all are Han), institutional status (almost all contain local agricultural *hukou*) and culture on the individual disaster preparedness. Moreover, this provided unique conditions to test the impact of place, especially the influence of geographic landforms, on the individual disaster preparedness of local rural residents.

### 2.2. Data Collection

A cross-sectional self-reported constructed questionnaire survey was administered to villagers aged 13 years old and above in the three stratified rural communities conducted over a week from 3 February 2018 to 10 February 2018, which was the week before the Chinese New Year festival. In this week, many of the villagers, who left the villages for employments, had come back to the rural communities for a family gathering. This is important for capturing and examining the impact of out-migration on individual disaster preparedness. Within each village, cluster sampling was used to avoid geographical or socioeconomic bias to ensure coverage of the entire community. Instead of a self-administered questionnaire survey, a face-to-face structured interview was adopted to make every rural participant fully understand our questionnaire, especially for adolescents, senior adults and low-educated groups. The survey in each village was conducted by a team of 24 young volunteers recruited from the universities from Xi’an, the capital city of Shaanxi province, and one local villager government officer from each village. Adolescents aged 13–17 were invited to participate in the survey since they become parts of the mainstream of disaster risk reduction in the rural communities while their parents migrated out for employments [56]. Ethics approval of the study was sought from the Human Subjects Ethics Sub-committee of The Hong Kong Polytechnic University (reference number: HSEARS20180323002). For the participants aged from 13 to 17, additional parental consent was sought.

### 2.3. Conceptual Framework and Measurement

This research adopted the ecological framework [57,58,59] to understand individual disaster preparedness of rural residents in Northwest China. The ecological framework was developed to examine individuals’ relationships within communities and the wider society [57]. This ecological framework assumed that there were multiple influences on individual behaviours at the intrapersonal, interpersonal, organizational, community and public policy levels, which, in turn, interact across different dimensions [59]. This study focused on individual disaster preparedness in relation to factors across five different dimensions: (1) place with different landforms, (2) individual sociodemographic factors, (3) family socioeconomic status, (4) hazard adaptions, and (5) community and neighbourhood influence.

#### 2.3.1. Dependent Variable: Individual Disaster Preparedness

This study adopted Federal Emergency Management Agency’s (FEMA) measurement appraisal of individual disaster preparedness [60,61] according to the local rural Chinese context. The individual disaster preparedness (InDP) was measured by five questions: (a) Disaster risk reduction (DRR) relevant meeting participation (Meeting) (0 = no, 1 = yes); (b) DRR relevant drill participation (Drill) (0 = no, 1 = yes); (c) DRR volunteer participation (Volunteer) (0 = no, 1 = yes); (d) awareness of nearest emergency shelter around the home (evacuation); and e) the self-reported level of disaster preparedness (self-reported level) (1 = I am not planning to do anything about preparing; 2 = I have not yet prepared, but I intend to in the next six months; 3 = I have not yet prepared, but I intend to do in the next month; 4 = I just recently began preparing; 5 = I have been prepared for at least the past six months). The score of InDP was the summation of meeting, drill and evacuation and the normalized score of the level with a range from 0 to 5.

InDP = Meeting + Drill + Volunteer + Evacuation + Normalized (Self-reported Level)

The normalization adopted a 0–1 scaling method:

$$Z_{i}=\frac{x_{i-\min\left(x\right)}}{\max\left(x\right)-\min\left(x\right)}$$

where *χ* = (*χ*_1_,…, *χ_n_*) and *Z_i_* is the *i*th normalized data.

#### 2.3.2. Independent Variables

(1) Place

Place in this research mainly referred to geographical location, landforms, and physical environments rather than the social and cultural contexts because the three selected villages located in the same county, which share the same language and dialect, ethnicity, institution and culture. The three selected villages located in three different geographical landforms: plain, plateau and mountain.

(2) Individual sociodemographic factors

The list of individual sociodemographic factors studied included: gender, age, education, marriage, occupation and main residential place in the last year. The option of occupation involved farming, non-agricultural work, schooling, housekeeper and others according to the local context. The main residential place was defined as the place where respondents had stayed for six months or above in the past year, which included the options of living in their villages, own towns, own counties/cities, own provinces (within Shaanxi Province), and another province (out of Shaanxi). The residents, whose main residential places in the last year were own province or another province, could be considered as out-migrants.

(3) Family socioeconomic status

The variable of family socioeconomic status included household income in the last year (RMB per year), housing construction (using reinforced concrete or not), housing size (square meters) and farmland size (*mu*, a Chinese unit for the area, which is about 666 ^2^⁄₃ square meters or approximately 0.165 acres).

(4) Hazard adaptions

Hazard adaptions were adapted from the study of community resilience by Cutter [28] to focus on hazard inherent and adaption, including hazard experience, knowledge, risk perception and self-efficacy when examining disaster preparedness of individuals. This study used droughts and floods, the most two frequent hazards in the local communities, to assess individual hazard adaptions. The list of variables included drought experience (0 = no, 1 = yes), flood experience (0 = no, 1 = yes), self-efficacy of government’s responsibility(it is the responsibility of government to assist once the disaster happens, 0 = no, 1 = yes), self-efficacy of individual responsibility (it is an individual responsibility, to take care of families during the first 72 h, once the disaster happens, 0 = no, 1 = yes), knowledge of drought (five-point Likert scale, 1 representing “do not know it at all”, 5 representing “fully understand it”), knowledge of flood (same scale with knowledge of drought), knowledge of DP (same scale with knowledge of drought), risk of drought (5-point Likert scale, 1 representing “do not have any risk”, 5 representing “have the highest risk”), and risk of a flood (same scale with the risk of drought).

(5) Community and Neighbourhood Influence

The influence by community and neighbourhood was mainly accessed by the DRR related activities or promotions organized in/by the community and whether there was any counterpart have started disaster preparedness in their neighbourhood [60,61]. The exact questions included whether there is any direct DRR related activity in the village (such as evacuation drills and disaster response skills training) (0 = no, 1 = yes), whether they were encouraged to create a family emergency response plan by the community/workplace/school (0 = no, 1 = yes), whether they were encouraged to participate in DRR related training by the community/workplace/school (0 = no, 1 = yes), and whether someone around had taken proactive steps to prepare for disasters (0 = no, 1 = yes). The score of community and neighbourhood influence was the sum of the above four questions with a range from 0 to 4.

### 2.4. Data Analyses

The first step was to have a descriptive statistic of the samples and independent variables. The second step was to capture the level of InDP in the three comparative villages. One-way ANOVA was applied to test the significance of the differences among the three villages. A post hoc test was applied in the ANOVA to determine the difference between the villages. The final step was to conduct a multi-level and multi-variable linear regression to further examine influences on the InDP, made across the five multi-dimension independent variables, which was based on the above conceptual framework and following models.

$$Individual\;disaster\;preparedness\;\approx \;\beta_{0}+\;\beta_{1}\times var_{1}+\ldots +\;\beta_{n}\times var_{n}$$

where *individual disaster preparedness* was the summation of the adequate disaster preparedness adopted, which ranged from 0 to 5; *var*_1_ to *var_n_* are variables in five different categories: (a) places; (b) individual sociodemographic factors; (c) family socioeconomic status; (d) hazard adaptions; and (e) community and neighbourhood influence.

This step was to identify the most critical and important predictors across different dimensions and observe the interaction between different dimensions when determining individual disaster preparedness. This study adopted listwise deletion to deal with missing data in regression analysis. Only cases with valid values for all variables were included in the analyses, which might cause the different sample size among the models with different intendent variables. This study conducted analyses using SPSS software, version 25.0 (IBM, New York, NY, USA), and set statistical significance at α = 0.05 two-tailed.

## 3. Findings

### 3.1. Descriptive Analyses

The cross-sectional self-reported survey collected N_1_ = 1080 valid individuals aged 13 years or above from N_2_ = 554 households in the three villages. Among the samples, 345 were from the plateau village (31.9%), 367 from the plains (34.0%) and 368 from the mountains (34.1%). The survey covered over half of the households in their three villagers (52%), which provided good representativeness.

Slightly more males (597 out of 1018, 55.3%) participated in our survey than did females (483 out of 1018, 44.7%) (Table 1). The mean age of participants (49 years old) was higher than the total population aged (40 years old), 13 years or above in Shaanxi Province [62]. Most respondents had junior secondary or below education levels, and the average education level in the mountain village was the lowest (mean = 7.5 years, standard deviation = 3.7). Over 90% of the participants were married and over half were farming (974 out of 1018, 90.2%). About one-quarter had non-agricultural employment in the last year (285 out of 1018, 26.4%), and this ratio was the highest in the mountain village (132 out of 368, 35.9%). Approximately 30% of the participants lived outside of their village, including towns, counties, own provinces or another province for six months or more in the past year. The proportion of living outside the village was highest in the mountain village (33.4%).

The household income in the last year was around RMB 20,596/year (about USD 3222/year), and the household income in the plateau village was the lowest (RMB 15,456/year, about USD 2418/year). However, the percentage of houses that used reinforced concrete was highest in the plateau village (31.6%) than the plains (16.3%) and mountain villages (2.4%). Many households had a large size of housing with a mean number of 115 m^2^ per household. The residents on the plains had the largest land size for agricultural production (mean = 5.9 mu, about 0.97 acres, standard deviation = 8.7) while the average size of farmland in the mountains was the smallest (mean = 1.1 *mu*, about 0.18 acres, standard deviation = 1.0).

Over three-quarters of the respondents in each village reported drought experience (904 out of 1018, 84.3%). However, only one-third of residents in the plateau village experienced floods, while over 90% of the residents in the plains village encountered a flood in their lives. In general, self-reported knowledge of floods (*r* = 0.133, *p* < 0.001) and disaster preparedness (*r* = 0.277, *p* < 0.001) had significant and positive correlated relationships with the level of InDP. The residents in the plains villages, proximal to the Wei River, reported a significantly higher flood risk level (mean = 4.54, standard deviation = 0.69) by using the self-report 5-point Likert scale (scoring 1 to 5) when compared to the villages in the plateau (mean = 3.24, standard deviation = 1.22) and mountains (mean = 4.01, standard deviation = 1.01). However, neither perceived risk of flood nor drought had a significant correlated relationship with the self-reported InDP. It was found that respondents, who perceived that it was government’s responsibility to assist the villagers once the disaster happen, reported a significant higher score of InDP (mean = 1.15, standard deviation = 1.17, *T-value* = −4.589, *p* < 0.001) than the resident who did not have such type of perception (mean = 0.71, standard deviation = 0.98). The respondents, who perceived that it was individual responsibility to take care of families during the first 72 h once the disaster happened, also reported a significant higher score of InDP (mean = 1.10, standard deviation = 1.15, *T-value* = −2.795, *p* < 0.01) than the resident who did not have such type of perception (mean = 0.66, standard deviation = 1.02). Further, the residents in the plains village (mean = 0.96, standard deviation = 1.21) and mountains (mean = 1.00, standard deviation = 1.27) reported a higher score of community influence than the plateau village (mean = 0.44, standard deviation = 0.89). There was a significant and positive correlated relationship between community influence and InDP (*r* = 0.399, *p* < 0.001).

### 3.2. Place and Individual Disaster Preparedness

**Figure 2:**
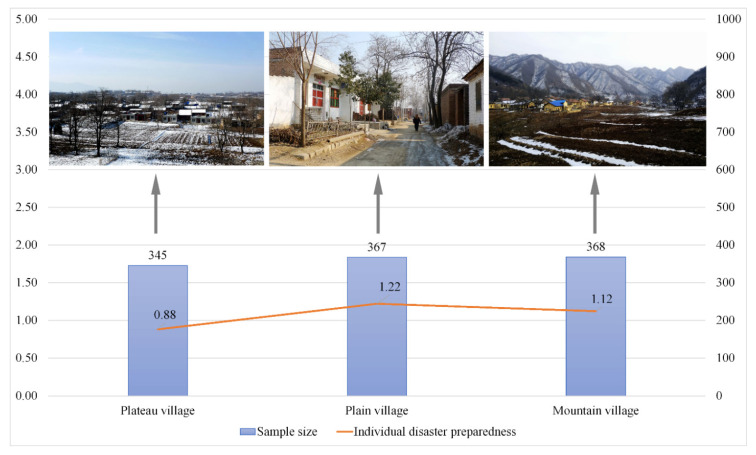
Level of self-reported individual and household disaster preparedness. Notes: (1) ANOVA test result of InDP among the three villages: F(2,1064) = 8.075, *p* < 0.001, with missing value = 13; (2) The photos were taken by the research team during the survey in February 2018.

Respondents in all three villages reported a low level of individual disaster preparedness when “5” was the highest score (mean = 1.07, standard deviation = 1.15). The plains village reported the highest level of InDP (mean = 1.22, standard deviation = 1.24), following by the mountain village (mean = 1.12, standard deviation = 1.13) and the plateau village (mean = 0.88, standard deviation = 1.04) (Figure 2). There was a significant effect of the geographic landforms on villager’s InDP for the three villages according to the ANOVA (F(2,1064) = 8.075, *p* < 0.001). Post-hoc comparisons using the Scheffe test indicated that the InDP in the plateau village significantly differed from the plains (*p* < 0.001) and mountain villages (*p* < 0.05). However, there is no significant difference in InDP between the plains and mountain villages (*p* = 0.488).

### 3.3. Multiple Regression Analyses

Multi-level and multi-variable linear regressions were performed to investigate the impact of place, individual sociodemographic factors, family socioeconomic status, hazard adaptions, and community influence on rural residents’ InDP. All the prediction models were statistically significant and accounted for 1.5% to 30.8% of the samples (Table 2).

The place was a significant factor influencing villagers’ InDP in Model 1 (Place, an unadjusted model before accounting for other factors), Model 2 (Place + Individual sociodemographic factors) and Model 3 (Place + Individual sociodemographic factors + Family socioeconomic status). Living in the plain village had a positive influence on the level of self-reported InDP. However, when considering disaster adaptions and community influence, the place was not a significant factor anymore (Models 4 and 5).

Gender was the only significant individual sociodemographic factor across the five different models. Females reported inverse relationships with the level of InDP. Without controlling for community influence, being a student was a significant and positive factor with InDP (Models 2–4). After adjusting the community influence, being a housekeeper had a significant and positive association with InDP (Model 5). Having non-agricultural work was not significant across the five models. However, being out-migrants, who had left their villages to live in other cities in their province for at least a half year in the last year was significant when considering community influence (Models 4 and 5, dummy variables of own province). Age had a significant and negative effect on InDP if the model did not involve family socioeconomic status and community influence (Models 2 and 3). However, the education level was not a significant factor when predicting rural residents’ InDP across different models (Models 2–5).

Among the factors of family socioeconomic status, standardized housing size was the only significant factor to predict InDP. Larger housing size yielded a higher score of InDP. Reinforced concrete housing, household income, and farmland size were not significant predictors for InDP (Models 3–5).

Having drought experience received a significant and inverse relationship with InDP. Self-efficacy of individual responsibility and knowledge of DP had a significant and positive predictive power on InDP (Models 4 and 5). Finally, community influence received the most substantial weight when examining disaster preparedness of individuals (Model 5).

## 4. Discussion

This study examined the individual disaster preparedness of rural residents in three drought-and-flood-prone villages with different landforms (plains, loess plateau, and mountains) via a cross-sectional self-report structured questionnaire survey conducted in Northwest China. An ecological framework was adopted to investigate the impacts of place, individual sociodemographic factors, family socioeconomic status, hazard adaptations, community and neighbourhood influences on rural residents’ individual disaster preparedness.

Significant differences were found in the self-reported individual disaster preparedness among the three villages with different geographic landforms. The residents in the plains village reported the highest level of individual disaster preparedness, which is located proximal to the Wei River which encountered a catastrophic flood disaster in 2003 [53]. However, the disaster experience of a flood was not a significant predictive factor according to the empirical models which were tested. Comparatively, the farmers in the plateau had encountered more and more severe drought in recent years due to climate change [63]. The experiences of drought had a significant but negative relationship with the individual disaster preparedness. That might be because there was seldom available resource for preparedness actions against droughts. The villagers might not be able afford the addition cost for drought preparedness [41]. Moreover, rural communities with different geographic landforms shared different types of resource, had different drives of economic development and encountered different types of disasters, even locating in the same district and very close to each other. For example, the farmland resource per household in the mountains was the smallest according to our observation, which might result in the highest percentage of out-migration for employment, among the three studied villages. This was also one of the interacts between multi-dimension influential factors when determining individual disaster preparedness, we found in northwest rural China. However, it was still difficult to tell whether specific landforms caused the differences of villagers’ individual disaster preparedness in three different rural communities because of the mixed impacts caused by other factors.

The impact of place on disaster preparedness was also significant when individual sociodemographic, household socioeconomic status, hazard adaptions were considered. However, with the adjustment of community influence, the factor of the place was not significant anymore in the model, which supported the notion that the community had a strong impact on one’s individual disaster preparedness. This could provide evidence that, in whichever community, DRR community participation and neighbourhood influence were highly important to enhance individual’s disaster preparedness, such as DRR- related meetings, drills, and publicity provided by the rural government committee or NGOs. No information on the DRR-related activities was captured in the three villages with different landforms. However, many previous studies had already addressed the significant and positive effect of DRR intervention activities on disaster preparedness in rural China, e.g., [16]. Therefore, it is necessary to conduct more research to develop more effective tailored DRR-related activities to enhance villagers’ individual disaster preparedness.

The giant groups of outmigration had profound impacts on the economic, social, cultural processes as well as community resilience in rural China [44,64]. Doing non-agricultural work was not a significant factor in one’s individual disaster preparedness according to the tested empirical model herein. However, the villagers who had lived away from their villages were more prepared for disasters. The villagers went out to work for increasing the household income, which can decrease household poverty vulnerability [64] and increase community resilience in rural areas [44]. At the same time, their disaster knowledge, risk perception and skills might have changed during their time living away from home. This also suggested that the rural residents, who had not left their communities to work, were the group of people who need DRR intervention the most to increase their disaster preparedness in the disaster-prone rural communities.

This study has some limitations. First, the survey design yielded cross-sectional data only. Cross-sectional data does not allow for the examination of the causal relationship between preparedness and the independent variables. Hence, future studies could replicate this study using longitudinal designs to have a better knowledge of the predictors of disaster preparedness of rural communities over time. Second, there might be possible sampling bias since the face-to-face surveys were conducted in the rural communities during the day. Surveys may have missed those rural residents who worked in the day or were out of village all year round. Third, the measurement of individual disaster preparedness could be expanded beyond the current interaction by using DRR-related meetings, drills, volunteer participation, awareness of the place of concentrated refuge, as well as the perceived stage of disaster preparedness. Finally, the estimation method we used did not allow for the specification of co-variances between the exogenous variables. This was deemed a drawback to our models used, as we did discover associations between the variables in the bivariate analyses. Finally, individual disaster preparedness might be potentially influenced by other types of factors, such as the disaster preparedness planning at community level, which has not been covered in this study.

## 5. Conclusions

In this study, we found that places with different landforms (plains, loess plateau and mountains) were a significant factor for individual disaster preparedness of the villagers in Northwest China when controlling individual sociodemographic and family socioeconomic factors. The level of preparedness in the plains was higher than both mountains and plateau. Moreover, the villagers who had out-migrated to work reported a higher level of disaster preparedness than did local villagers. In addition, the community and neighbourhood played an important role in determining individual disaster preparedness. This article contributed empirically and practically to the literature about the individual disaster preparedness in the rural communities and their determinants. This study recommends that the community-based DRR program should be tailored according to the local context, including the geographic landforms, hazard experience, and population diversity. Further research is needed to have more longitudinal observations and nuanced examinations of individual disaster preparedness, especially in under-resourced rural communities, to develop more relevant policy and disaster preparedness educational programs for these communities.

## Figures and Tables

**Table 1:** Descriptive analyses of the samples and individual disaster preparedness.

Dimension	Independent Variables		Plateau (*n* = 345)	Plains (*n* = 367)	Mountains (*n* = 368)	Total Samples (*n* = 1018)	InDP *M* (*SD*)	*t*-Test/ANOVA/Correlation Analyses ^
			*N* (%)/ *M* (*SD*)	*N* (%)/ *M* (*SD*)	*N* (%)/ *M (SD*)	*N* (%)/ *M* (*SD*)		
**Individual sociodemographic factors**	Gender	*Male*	193 (55.9%)	205 (55.9%)	199 (54.1%)	597 (55.3%)	1.15 (1.18)	*t* = 2.182, *p* < 0.05
		*Female*	152 (44.1%)	162 (44.1%)	169 (45.9%)	483 (44.7%)	0.99 (1.11)	
	Marriage	*Married*	323 (93.6%)	337 (91.8%)	314 (85.3%)	974 (90.2%)	1.02 (1.10)	*t* = −5.193, *p* < 0.001
		*Unmarried*	22 (6.4%)	30 (8.2%)	54 (14.7%)	106 (9.8%)	1.62 (1.44)	
	Occupation	*Farming*	229 (66.4%)	225 (61.3%)	155 (42.1%)	609 (56.4%)	0.95 (1.03)	F(4,1062) = 14.085, *p* < 0.001
		*Non-agricultural*	63 (18.3%)	90 (24.5%)	132 (35.9%)	285 (26.4%)	1.15 (1.20)	
		*Schooling*	10 (2.9%)	16 (4.4%)	26 (7.1%)	52 (4.8%)	2.16 (1.33)	
		*Housekeeper*	32 (9.3%)	20 (5.4%)	37 (10.1%)	89 (8.2%)	1.12 (1.25)	
		*Others*	11 (3.2%)	16 (4.4%)	18 (4.9%)	45 (4.2%)	1.05 (1.28)	
	Residential location in the last year	*Own village*	264 (76.5%)	286 (77.9%)	245 (66.6%)	795 (73.6%)	1.01 (1.10)	F(4,1062) = 4.412, *p* < 0.01
		*Own town*	6 (1.7%)	3 (0.8%)	43 (11.7%)	52 (4.8%)	1.26 (1.24)	
		*Own County/* *City*	10 (2.9%)	23 (6.3%)	26 (7.1%)	59 (5.5%)	1.29 (1.35)	
		*Own Province*	40 (11.6%)	27 (7.4%)	35 (9.5%)	102 (9.4%)	1.23 (1.24)	
		*Another Province*	25 (7.2%)	28 (7.6%)	19 (5.2%)	72 (6.7%)	1.05 (1.22)	
	Mean of age (years)		52.5 (13.9)	49.7 (15.1)	45.1 (15.7)	49.0 (15.3)	-	*r* = −0.180, *p* < 0.001
	Education level (years)		8.5 (3.3)	8.8 (2.9)	7.5 (3.7)	8.3 (3.3)	-	*r* = 0.114, *p* < 0.001
**Family socioeconomic status**	Reinforced concrete housing	*No*	236 (68.4%)	307 (83.7%)	359 (97.6%)	902 (83.5%)	1.11 (1.14)	*r* = −0.089, *p* < 0.01
		*Yes*	109 (31.6%)	60 (16.3%)	9 (2.4%)	178 (16.5%)	0.90 (1.20)	
	Household income in last year (RMB)		15,456 (18,789)	25,099 (40,554)	20,939 (25,335)	20,596 (30,065)	-	*r* = 0.037, *p* = 0.229
	Housing size (m^2^)		120.3 (40.8)	120.0 (41.1)	104.1 (38.9)	114.7 (41.0)	-	*r* = 0.023, *p* = 0.454
	Farmland size (*mu*)		3.5 (1.3)	5.9 (8.7)	1.1 (1.0)	3.5 (5.6)	-	*r* = 0.023, *p* = 0.454
**Hazard Adaptions**	Drought experience	*No*	36 (10.4%)	47 (13.0%)	85 (23.3%)	168 (15.7%)	1.37 (1.26)	*t* = 3.605, *p*<0.001
		*Yes*	309 (89.6%)	314 (87.0%)	281 (76.8%)	904 (84.3%)	1.01 (1.12)	
	Flood experience	*No*	228 (66.1%)	27 (7.5%)	146 (39.9%)	401 (37.4%)	0.97 (1.11)	*t* = −2.189, *p*<0.05
		*Yes*	117 (33.9%)	334 (92.5%)	220 (60.1%)	671 (62.6%)	1.13 (1.17)	
	Government’s responsibility	*No*	104 (30.1%)	47 (12.4%)	26 (7.1%)	177 (16.4%)	0.71 (0.98)	*t* = −4.589, *p*<0.001
		*Yes*	241 (69.9%)	320 (87.2%)	342 (92.9%)	903 (83.6%)	1.15 (1.17)	
	Individual responsibility	*No*	12 (3.5%)	20 (2.0%)	25 (6.8%)	57 (5.3%)	0.66 (1.02)	*t* = −2.795, *p*<0.01
		*Yes*	333 (96.5%)	347 (94.6%)	343 (93.2%)	1023 (94.7%)	1.10 (1.15)	
	Knowledge of drought (1–5)		3.16 (1.31)	3.18 (1.23)	2.87 (1.20)	3.07 (1.25)	-	*r* = 0.021, *p* = 0.487
	Knowledge of flood (1–5)		2.13 (1.16)	3.36 (1.27)	2.79 (1.36)	2.77 (1.36)	-	*r* = 0.133, *p* < 0.001
	Knowledge of DP (1–5)		1.83 (0.96)	2.22 (1.05)	2.04 (0.89)	2.03 (0.97)	-	*r* = 0.277, *p* < 0.001
	Risk of drought (1–5)		4.48 (0.75)	4.15 (0.97)	3.95 (1.03)	4.19 (0.95)	-	*r* = −0.001, *p* = 0.984
	Risk of flood (1–5)		3.24 (1.22)	4.54 (0.69)	4.01 (1.01)	4.03 (1.09)	-	*r* = 0.066, *p* = 0.055
**Community**	Community and Neighbourhood Influence (0–4)		0.44 (0.89)	0.96 (1.21)	1.00 (1.27)	0.81 (1.17)	-	*r* = 0.399, *p* < 0.001

Note: ^, T-test was conducted for the binary independent factors; ANOVA test was conducted for the categorical independent variables with more than two groups; Spearman correlated analyses was conducted for the continuous independent variables. M, mean; SD, standard deviation.

**Table 2:** Results of multiple regression analyses on individual disaster preparedness.

	Model 1	Model 2	Model 3	Model 4	Model 5
**Place (Ref. Plateau)**					
Plains	0.068 (0.017) ***	0.057 (0.017) **	0.050 (0.018) **	0.048 (0.027)	0.019 (0.025)
Mountains	0.047 (0.017) **	0.026 (0.018)	0.030 (0.020)	0.013 (0.027)	−0.008 (0.024)
**Individual sociodemographic factors**					
Female		−0.038 (0.015) *	−0.037 (0.015) **	−0.046 (0.017) **	−0.046 (0.016) **
Unmarried		−0.003 (0.032)	0.004 (0.032)	0.016 (0.036)	0.010 (0.033)
Occupation (Ref. Farming)					
Non-agricultural		0.014 (0.025)	0.024 (0.025)	0.010 (0.029)	0.009 (0.026)
Schooling		0.158 (0047) **	0.157 (0.047) **	0.113 (0.054) **	0.072 (0.049)
Housekeeper		0.045 (0.026)	0.045 (0.026)	0.088 (0.033) **	0.060 (0.030) **
Others		0.011 (0.035)	0.000 (0.036)	−0.006 (0.042)	−0.023 (0.038)
Residential location in the last year (Ref. own village)					
Own town		0.004 (0.037)	−0.014 (0.037)	0.017 (0.043)	−0.014 (0.040)
Own county/city		0.010 (0.036)	0.005 (0.037)	−0.030 (0.042)	−0.031 (0.038)
Own province		−0.019 (0.031)	−0.029 (0.031)	−0.064 (−0.036)	−0.071 (0.033) **
Another province		−0.048 (0.036)	−0.058 (0.036)	−0.055 (0.040)	−0.064 (0.036)
Age (years)		−0.002 (0.001) **	−0.002 (0.001) **	−0.001 (0.001)	−0.001 (0.001)
Education (years)		0.004 (0.002)	0.003 (0.002)	0.002 (0.003)	0.001 (0.002)
**Family socioeconomic status**					
Reinforced concrete housing (Ref. no)			−0.034 (0.020)	−0.026 (0.023)	−0.016 (0.021)
Standardized household income			−0.007 (0.133)	−0.048 (0.152)	−0.004 (0.138)
Standardized housing size			0.218 (0.066) **	0.145 (0.077)	0.118 (0.070)
Standardized farmland size			0.131 (0.109)	−0.023 (0.123)	−0.019 (0.112)
**Hazard adaptions**					
Drought experience				−0.065 (0.027) **	−0.052 (0.024) **
Flood experience				0.037 (0.023)	0.024 (0.021)
Government’s responsibility				0.037 (0.024)	0.014 (0.022)
Individual responsibility				0.103 (0.034) **	0.082 (0.031) **
Knowledge of drought				0.009 (0.010)	0.005 (0.009)
Knowledge of Flood				0.001 (0.010)	0.003 (0.009)
Knowledge of DP				0.052 (0.009) ***	0.035 (0.008) ***
Risk of drought				0.010 (0.010)	0.012 (0.009)
Risk of flood				−0.018 (0.009) *	−0.011 (0.008)
**Community and neighbourhood influence**					0.081 (0.006) ***
N	1066	1055	1049	780	778
R^2^	0.015	0.076	0.090	0.161	0.308
ANOVA	F(2,1064) = 8.075, *p* < 0.001	F(14,1041) = 6.079, *p* < 0.001	F(18,1031) = 5.695, *p* < 0.001	F(27,753) = 5.350, *p* < 0.001	F(28,750) = 11.914, *p* < 0.001

Notes: Coefficients and standardized error (in brackets) were reported for each independent variable; *, *p* < 0.05; **, *p* < 0.01; ***, *p* < 0.001.

## Data Availability

The data presented in this study are available on request from the corresponding author. The data are not publicly available due to the agreement with survey participants.
